# Alzheimer’s disease pathologies are pathogen‐driven

**DOI:** 10.1002/alz.087008

**Published:** 2025-01-03

**Authors:** Vanesa R. Hyde, Chaoming Zhou, Juan R. Fernandez, Leonardo D'Aiuto, Or A. Shemesh

**Affiliations:** ^1^ The University of Pittsburgh, Pittsburgh, PA USA

## Abstract

**Background:**

Alzheimer’s disease (AD) is diagnosed via postmortem detection of extracellular amyloid beta (Aβ) plaques or oligomers and intracellular hyperphosphorylated tau. These canonical pathologies are key players in AD etiology. A complementary line of research suggests that common human pathogens serve as the initial seeding agents which facilitate the pathologies of AD.

**Method:**

Low (n = 8), mild (n = 9), and advanced (n = 10) Alzheimer’s disease brain samples were subjected to expansion microscopy (ExM), a tissue manipulation method which enables the imaging of biological samples at 40nm resolution. For our purposes, we used a novel version of ExM, decrowding expansion pathology (dExPath), which exposes inaccessible protein epitopes to antibody staining while enabling super resolution imaging. Observations from these human brain samples were then modeled using human iPSC neuronal 3D brain organoids and primary rodent cultures.

**Result:**

Using dExPath, we visualized pathogen‐related proteins within our Alzheimer’s disease human brain samples. We discovered a clear colocalization between AD pathologies and pathogen‐derived proteins. When utilizing human brain organoids and rodent cultures as model systems for the human brain, we used pathogen infection to replicate the AD pathologies observed in the AD human brain.

**Conclusion:**

Our results suggest that (1) neurons exposed to pathogens produce human AD pathologies and (2) AD pathologies may be antimicrobial. With these important roles in mind, our continued research focuses on isolating the mechanisms by which AD pathologies arise in response to common pathogens.

